# The Health of the City

**Published:** 1854-11

**Authors:** 


The following articles, originally written for the Buffalo Commercial Advertiser, are thought to have sufficient medical interest to warrant their transfer to our pages: H.

*The Health of the City; Past, Present, and Prospective.*—The last case of cholera of the season has occurred. The weekly report of deaths has dwindled to one-fourth the proportions of six weeks ago; one case only appearing in last week's record—a poor German, reported by his physician and fellow-countryman as "*in collapsus—nothing extra;*" as if it were "nothing extra" to close the list of that long array who have fallen under the shadow of the cholera's black wing.

"The blessed seals
Which close the pestilence"

are renewed, and we may look back now, and count the dead, wounded, and missing, and notice where the fray was thickest.

We wish to call the attention of our readers to the statistics we have gathered for this article, believing that they teach a lesson of warning and encouragement alike. We have collected them at an expense of no little time, and labor, and headache. They are not yet as complete as we wish, and intend to make them; but time presses, and the season when our citizens feel an interest in cholera statistics is rapidly passing away. We give them as they are, vouching for their accuracy so far as they go. They are not confined to cholera alone, but embrace, also, the allied diseases, cholera morbus, cholera infantum, diarrhœa, and dysentery. Any deficiencies or sources of error will be stated. During the months of June, July, August, and September, there have died in the city of Buffalo of

| | | |
|---|---|---|
| Cholera, | 545 | cases. |
| Cholera Morbus, | 25 | " |
| Cholera Infantum, | 157 | " |
| Diarrhœa, | 115 | " |
| Dysentery, | 69 | " |
| Total, | 911 | " |

Of the cholera cases 328 were males, 210 females, and in 7 the sex is unknown.

The localities of these deaths were ascertained in nearly all cases, so that the city may be divided into districts, and the mortality of each be set by itself. But there is a manifest impropriety in enumerating a portion of the cases as belonging to the district in which they died. Those dying at the Hospital of the Sisters of Charity, were collected from all parts of the city. In Dr. Newman's report, which is now preparing, these cases will be assigned to their proper localities. At the Poorhouse, also, there was a total of 84 deaths during July and August; 61 of them dying of cholera. The Poorhouse, then, has a fair claim to be a district by itself, inasmuch as it is removed from all the causes which may act in the city. Taking out, then, the deaths at the Poorhouse, the Hospital, those occurring in unknown localities, those of emigrants and travelers, and 6 cases occurring on streets which we cannot find, we have left 378 cases of cholera, and nearly all the cases of cholera morbus, cholera infantum, diarrhœa, and dysentery, which we can assign correctly to their respective districts.

For purposes of comparison we have divided the city into 9 districts.

District, No. 1, is bounded by Terrace, Main st., Lake Erie, and Erie st. The resident population is not large, the streets are nearly all paved, but, owing to the commerce transacted there, always dirty.

In District No. 1, there died of

| | |
|---|---|
| Cholera, | 33 |
| Cholera Morbus, | 2 |
| Cholera Infantum, | 6 |
| Diarrhœa, | 6 |
| Dysentery, | 1 |
| Total, | 48 |

District No. 2, is bounded north by Main and Hamburgh street canal, and a line extending thence due east to the city line, east by city line, south by Lake Erie, and west by Main street.

In District No. 2, there died of

| | |
|---|---|
| Cholera, | 37 |
| Cholera Morbus, | 1 |
| Cholera Infantum, | 8 |
| Diarrhœa, | 8 |
| Dysentery, | 10 |
| Total, | 64 |

District No. 3, is bounded north by Clinton, east by Michigan, south by Hamburgh street canal, and west by Main street.

In District No. 3, there died of

| | |
|---|---|
| Cholera, | 14 |
| Cholera Morbus, | 0 |

| | |
|---|---|
| Cholera Infantum, | 7 |
| Diarrhœa, | 4 |
| Dysentery, | 5 |
| Total, | 30 |

District No. 4, is bounded on the north by Clinton, east by city line, south by Hamburgh street canal and a line drawn due east to city line, and west by Michigan street.

In District No. 4, there died of

| | |
|---|---|
| Cholera, | 63 |
| Cholera Morbus, | 0 |
| Cholera Infantum, | 20 |
| Diarrhœa, | 7 |
| Dysentery, | 4 |
| Total, | 94 |

This District comprises the mortality on South Division street, and in the neighborhood of the Hydraulics.

District No. 5, is bounded on the north by the city line, (not including Poorhouse,) east by Michigan street, south by Clinton, and west by Main.

In District No. 5, there died of

| | |
|---|---|
| Cholera, | 15 |
| Cholera Morbus, | 1 |
| Cholera Infantum, | 14 |
| Diarrhœa, | 10 |
| Dysentery, | 5 |
| Total, | 45 |

District No. 6, comprises the German portion of the city, and is bounded north and east by the city line, south by Clinton street, and west by Michigan street.

In District No. 6, there died of

| | |
|---|---|
| Cholera, | 177 |
| Cholera Morbus, | 18 |
| Cholera Infantum, | 65 |
| Diarrhœa, | 55 |
| Dysentery, | 26 |
| Total, | 341 |

District No. 7, is bounded on the north by the city line, east by Main street, south by the Terrace, and west by Delaware street.

In District No. 7, there died of

| | |
|---|---|
| Cholera, | 7 |
| Cholera Morbus, | 0 |
| Cholera Infantum, | 9 |
| Diarrhœa, | 5 |
| Dysentery, | 1 |
| Total, | 22 |

District No. 8, is bounded north by Niagara street, east by Terrace, south by Lake Erie, and west by Prospect Hill.

In District No. 8, there died of

| | |
|---|---|
| Cholera, | 20 |
| Cholera Morbus, | 2 |
| Cholera Infantum, | 6 |
| Diarrhœa, | 8 |
| Dysentery, | 3 |
| Total, | 39 |

District No. 9, is bounded north and west by the city line, south by Niagara, and east by Delaware streets, comprising Black Rock.

In District No. 9, there died of

| | |
|---|---|
| Cholera, | 12 |
| Cholera Morbus, | 0 |
| Cholera Infantum, | 8 |
| Diarrhœa, | 6 |
| Dysentery, | 3 |
| Total, | 29 |

From these data we may arrive at some conclusions as to the healthfulness of our city proper.

If we link together Districts No. 3, 5, 7, 8, and 9, we have, in a district beginning at Main street bridge, bounded by Main to Terrace, Terrace to Delaware, Delaware to Niagara, Niagara to new city line, city line to Michigan, Michigan to Hamburgh street canal, and canal to Main, including Black Rock, comprising a population of about 45,000 souls, only 165 deaths from the diseases on our list, of which 68 only were from cholera. During four months of the prevalence of cholera, only 1 in 273 of this population has perished of any disease allied to it.

Now let us connect Districts 1, 2, and 8, making a cordon around the city thus: We start from the lake at the penitentiary, and go to Niagara, down Niagara to Delaware, down Delaware to Terrace, Terrace to Main,

Main to Hamburgh street canal, and thence east to city line, making a long, crooked district, following the curves of river, lake, and creek, and comprising all the lowest portion of our town; hardly any point, except the Penitentiary, attaining an elevation of fifteen feet above the usual water mark.

In this district we have 151 deaths by our group of diseases, in a population which does not exceed 10,000—being 1 in 66 of the population.

Place, now, the two remaining districts, Nos. 4 and 6, together, and we have all that portion of the city east of Michigan street, and north of the Hamburgh street canal; comprising a population, mostly German, which is usually estimated at 25,000

In this district there died of

| | |
|---|---|
| Cholera, . . . . . . . . . | 240 |
| The other diseases, . . . . . . . . | 197 |
| Total, . . . . . . . . | 437 |

This would give a ratio of deaths of 1 in 57, an alarmingly large proportion.

But there is a District, No. 10, not included in our previous calculations—the Erie County Poorhouse. It is said by the Superintendent of the Poor, that on the first day of July there were 270 inmates in this institution. During July and August, (we have no account of September,) 84 persons died in the house. Of these, 61 died of cholera, and some of diseases not included in our list. Classing them altogether, we have a mortality of 1 in $3\frac{21}{100}$, occurring in two months. At this rate the whole population of that palace of the poor would die off in a little more than six months.

We have already extended our article beyond our purpose. In conclusion, the reader will see that our city presents the anomaly of being, at once, the healthiest and the most sickly of any American town. The larger mass—nearly the whole of the American population—live in a security as to life and health, which can hardly find a parallel in any other place of its size. For a city like this to pass through a cholera season with so trivial a loss is wonderful. But let any one turn to the dirty, unpaved and crooked streets, in which our foreign population dwell, and he will wonder that so many survive. A calculation of the number of deaths upon the unpaved streets, would show how much drainage and dryness have to do with health.

We do not think it necessary to say much "by the way of improvement," except that our statistics have been faithfully compiled from the books in the city clerk's office, aided by our own observation.

In a second article we shall consider the conclusions to be drawn from these facts.

(*Art.* 2.) Were the surface of the Ohio Basin to remain unruffled by a keel, or unshadowed by a sail, for all time to come, its construction would have been a paying operation for the city of Buffalo.

The earth removed in its excavation has raised the street level of a whole district, from six to twelve feet, has capacitated it for drainage, and made of a dirty swamp a dry and habitable portion of the city. The neighborhood of the Elk Street Market has, in previous years, been *the* cholera district of the city. In 1849 all the causes of disease were in operation there; in 1852 they were but partially removed; in 1854 they had nearly passed away. The record of the cholera for those years runs parallel with the condition of the streets.

The central portion of the city possesses all the natural advantages of declivity and dryness of soil which tend to make a healthy town. Added to this fact, it is paved with a pavement far better than most cities can boast, and in the houses of its inhabitants are found as much comfort and abundance as can be found anywhere else in the same area. The effect of these causes is seen in the record of its mortality. It is true that cholera has entered a very few of the best regulated homes, but these cases are but proofs of the existence of a cholera atmosphere over *all* the city; while the exemption from fatal disease which has marked the great body of its citizens, has proved with equal clearness that dryness, cleanliness, and comfort, may ward off the effects of the cholera predisposition in nearly every case. Peculiar constitutions, personal imprudences, and undetected local causes, will furnish some apparent exceptions to this rule.

The same good health which marks our central and northwestern district may, and doubtless will be yet extended to the northeastern part of the city. This district, so severely visited this year, possesses the capabilities for drainage, dryness and the other means of health in a much greater degree than did the region about the market ten years ago. It only remains to apply them, and Buffalo will become, what the energy of its citizens far more than its natural qualities is destined to make it—one of the most healthy towns on the continent.

We have a right to expect this, when we look at the single fact that of the streets upon which cholera occurred this season, (so far as ascertained,) 28 were paved, and 62 were unpaved; while this fact is made still more important when we add that of 378 deaths, 230 happened upon unpaved, and 148 upon paved streets. To give force to this footing, it should be recollected that a very large part of the population lived upon paved streets —of the 148 deaths, 56 happened on Genesee street, which, though paved, is so intersected by unpaved streets, and so uncleanly in itself, as to do away with the usefulness of the pavement in some degree. Seneca, Exchange and South Division streets, upon which 44 deaths occurred east of Michigan street, are also much intersected by unpaved streets, and at the Hydraulics subjected to powerful local malarial influences.

As an illustration of the effect of sanitary reform upon the health of towns, the following facts may serve a good purpose, and perhaps do away with some of that foolish cant that talks about the degeneracy of the age, and the robust health and long lives of our ancestors. Macaulay (History of England) says, that "In the year 1685, not accounted a sickly year, more than one in twenty of the inhabitants of the capital died; at present only one in forty dies annually." Dr. Simpson "states that in the year 1786, the yearly rate of mortality in the whole of England and Wales, was one in forty-two; in 1801 it was one in forty-seven, and in 1831 it had diminished to one in forty-eight."

In order to arrive at the average mortality of Buffalo in its present condition, we must take the first six months as a standard of the year in a common season. Inspector Downing, of New York, makes these six months furnish, in 1852, forty-eight per cent., and in 1853, forty-five per cent. of the deaths of the year. Assuming the lesser figure as the probable per centage of this year, we had in 80,000 souls, 972 deaths during the first half of the year from all causes. By a simple proportion we may say what the number of deaths for the remaining six months of the year *should be*—as 45 is to 972, so is 55 to 1188. We have already had 1398 deaths in the first three months of the last half of the year; it is evident, therefore, that we have already overrun the usual number for a year.

But assuming that the average derived from the first six months is the usual and fair one for a series of years, we have one death in thirty-seven as the annual mortality. As London, which is said to be the most healthy city in the world, has an annual mortality of one in forty, the comparison is largely in favor of Buffalo as compared with other American cities. Estimating the number of deaths for the remaining quarter at 518, or 24 per cent. of a common year, the mortality of 1854 will be 2866, or one in twenty-eight of the population. This is not above the usual ratio of many towns considered healthy.

It would be wrong to conclude a statistical article on the diseases of Buffalo, without referring to the mortality at the Erie County Poorhouse. The opinions which were expressed last summer in our columns relative to the causes of that appalling visitation, remain unchanged. We have shown in the figures of this article that local causes are required for the development of cholera in an epidemic form. Now what are the causes in this instance? If, as we are told by the Superintendents, the house is well constructed, and the diet good and sufficient, *why* do they have cholera at such a rate? Admitting their statements, it passes our comprehension, and is beyond the reach of reason or statistics.

The annual report of the Superintendents for the year ending Oct. 1854, shows, 170 deaths in an average population of 378. This gives a mortality of 1 in $2\frac{22}{100}$, but if we take the more liberal, though less fair estimate, based upon the ratio of deaths to persons relieved, (though many of them may not have been a week in the house,) we have a mortality of 1 in $9\frac{16}{100}$. Let it be recollected that this is the ratio of deaths in a house which receives lunatics, idiots, cripples, and destitute poor, in much larger proportion than the sick proper, and that a large proportion of its inmates remain but a short time. Of the 281 inmates now there, it is said that 105 are more or less ill. This is a large proportion of sick for an almshouse, but many of them are but slightly ill, and 175 are called in health. This statement may give some idea of the difference between the Erie County Poorhouse and the other institutions we wish to compare with it, where none but the sick are admitted.

In New York Hospital the mortality is 1 in $11\frac{1}{8}$.

In Charity Hospital of New Orleans it is 1 in 11.

In Pennsylvania Hospital it is 1 in $11\frac{87}{100}$.

In Quarantine Hospital, (N. Y.,) during the ship fever of 1847, it was 1 in 16.

In Erie County Poorhouse it is 1 in $9\frac{16}{100}$.

Is it possible that a more fatal class of cases reaches our Poorhouse than enters the hospitals of our largest cities? We have purposely placed the Poorhouse in comparison with these particular institutions, as they cannot be objected to as unfair; their averages being based on long periods of years, with the exception of the Quarantine Hospital. But if we choose to take isolated years in other institutions, we could mention the Bellevue Hospital, (N. Y.,) in 1848, where 369 died out of 8,792 admissions, 1,226 of which were cases of ship fever, making a ratio of only 1 in 23.

Six years ago, a distinguished physician of this city asserted that the mortality at our Poorhouse was greater than in any hospital or ship fever sheds in the country. He proved it by facts and figures like our own, and the assertion is as true now as then. The whole policy of the Poorhouse is niggardly and mean. Cheap provisions, cheap doctors, cheap nurses, cheap medicines, cheapness everywhere is the rule, forgetting that higher policy which finds true economy in a humane liberality.

